# Ectopic Expression of FVIII in HPCs and MSCs Derived from hiPSCs with Site-Specific Integration of *ITGA2B* Promoter-Driven *BDDF8* Gene in Hemophilia A

**DOI:** 10.3390/ijms23020623

**Published:** 2022-01-06

**Authors:** Junya Zhao, Miaojin Zhou, Zujia Wang, Lingqian Wu, Zhiqing Hu, Desheng Liang

**Affiliations:** Center for Medical Genetics, School of Life Sciences, Central South University, Changsha 410078, China; zhaojunya@sklmg.edu.cn (J.Z.); zhoumiaojin@sklmg.edu.cn (M.Z.); wangzujia@sklmg.edu.cn (Z.W.); wulingqian@sklmg.edu.cn (L.W.)

**Keywords:** hemophilia A, platelet-targeted strategy, nonviral vector, rDNA locus, hematopoietic progenitor cell, mesenchymal stem cell

## Abstract

Hemophilia A (HA) is caused by mutations in the coagulation factor VIII (FVIII) gene *(F8)*. Gene therapy is a hopeful cure for HA; however, FVIII inhibitors formation hinders its clinical application. Given that platelets promote coagulation via locally releasing α-granule, FVIII ectopically expressed in platelets has been attempted, with promising results for HA treatment. The B-domain-deleted *F8* (*BDDF8*), driven by a truncated *ITGA2B* promoter, was targeted at the ribosomal DNA (rDNA) locus of HA patient-specific induced pluripotent stem cells (HA-iPSCs). The *F8*-modified, human induced pluripotent stem cells (2bF8-iPSCs) were differentiated into induced hematopoietic progenitor cells (iHPCs), induced megakaryocytes (iMKs), and mesenchymal stem cells (iMSCs), and the FVIII expression was detected. The *ITGA2B* promoter-driven *BDDF8* was site-specifically integrated into the rDNA locus of HA-iPSCs. The 2bF8-iPSCs were efficiently differentiated into 2bF8-iHPCs, 2bF8-iMKs, and 2bF8-iMSCs. FVIII was 10.31 ng/10^6^ cells in lysates of 2bF8-iHPCs, compared to 1.56 ng/10^6^ cells in HA-iHPCs, and FVIII was 3.64 ng/10^6^ cells in 2bF8-iMSCs lysates, while 1.31 ng/10^6^ cells in iMSCs with CMV-driven *BDDF8*. Our results demonstrated a high expression of FVIII in iHPCs and iMSCs derived from hiPSCs with site-specific integration of *ITGA2B* promoter-driven *BDDF8*, indicating potential clinical prospects of this platelet-targeted strategy for HA gene therapy.

## 1. Introduction

Hemophilia A (HA) is an X-linked recessive genetic disorder that affects approximately 1 in 5000 newborn boys. The clinical symptoms of HA patients are mainly spontaneous joint and muscle bleeding, or traumatic bleeding, leading to disability; it can even be life-threatening due to intracranial hemorrhage [1]. At present, there is no cure, and the main clinical treatment is replacement therapy with intravenous injections of plasma-derived or recombinant coagulation factor VIII (FVIII) protein. However, as the half-life of FVIII in the plasma is only 12 h [2], patients require repeated infusions, which makes this treatment very costly and brings a heavy economic and psychological burden to them and their families. Currently, the novel EHL-factors and the non-factor therapies are reducing the burden of the life-long treatment without impacting the long-term therapeutic solution of gene therapy in hemophilia [3]. A major complication is the formation of FVIII inhibitor, which affects approximately 25% to 30% of HA patients, decreasing its coagulation efficacy [4,5].

Gene therapy provides a promising option for HA [6]. As a slight increase in the coagulation activity of plasma FVIII can significantly alleviate the clinical bleeding symptoms, HA has been considered as a suitable disease for gene therapy [7]. Currently, gene therapy for hemophilia based on adeno-associated virus (AAV) vectors has been advanced to clinical trials [8,9]. However, there are still some challenges for the AAV-based gene therapy. About 30–60% of the population have antibodies against different serotypes of AAV capsids, and different serotypes have cross-immunity, which greatly limits its wide application [10]. Moreover, it is worth noting that there is a risk of insertion mutation in the use of viral elements. Recently, in the long-term follow-up study of HA gene therapy based on AAV, the integration of AAV vectors near the genes that control cell growth in HA model dog hepatocytes was found, which has a potential risk of inducing tumors [11].

To circumvent some concerns, reduce the potential risks, and provide a choice for the HA patients that have antibodies against AAV capsids, an ex vivo gene therapy strategy has been actively studied [12,13]. This strategy can select cells without off-target mutations via whole-genome sequencing analysis and provide a reliable cell source, possessing higher safety. Induced pluripotent stem cells (iPSCs) have become ideal targets for ex vivo gene therapy due to their unlimited proliferation ability and multidirectional differentiation potential.

Immune response has always been a key problem in HA gene therapy. 25–30% of HA patients produce FVIII inhibitory antibodies, resulting in ineffective therapy. The ectopic expression of FVIII in platelets is an effective strategy that reduces the immune response against FVIII [14,15]. Once blood vessels are damaged, platelets will immediately gather at the damaged site and secrete FVIII to coagulate the damaged vessels. The genetically modified FVIII function acts directly from platelets to the bleeding site without entering the circulatory system, thus reducing the risk of producing FVIII inhibitors. Some studies have used bone marrow-derived hematopoietic stem cells (HSCs) modified using lentivirus to achieve the restoration of the FVIII function [16]. However, the HSCs isolation procedure is traumatic for patients and there is an insertional risk for lentivirus. In recent decades, there have been reports of hematopoietic progenitor cells (HPCs) generated from human embryonic stem cells and human iPSCs (hiPSCs), and harvested megakaryocytes (MKs) and platelets by further differentiation in vitro [17,18]. Further, some studies have shown that lipogenic mesenchymal stem cells can differentiate into MKs in vitro [19]. Differentiation of hiPSCs into HPCs and mesenchymal stem cells (MSCs) may be an efficient method to generate an infinite source of genetically modified targeted cells with uniform quantity and quality.

In this study, the truncated *ITGA2B* promoter-driven B-domain-deleted *F8* (*BDDF8*) named *2bF8* was targeted at the ribosomal DNA (rDNA) locus of HA patient-specific iPSCs (HA-iPSCs), which were subsequently differentiated into HPCs and MSCs. The ectopically expressed FVIII was validated in HPCs and MSCs. This strategy may provide an innovative approach for platelet-targeted gene therapy for HA.

## 2. Results

### 2.1. Construction of Nonviral Targeting Vector minipHrn-2bF8

**Figure 1:**
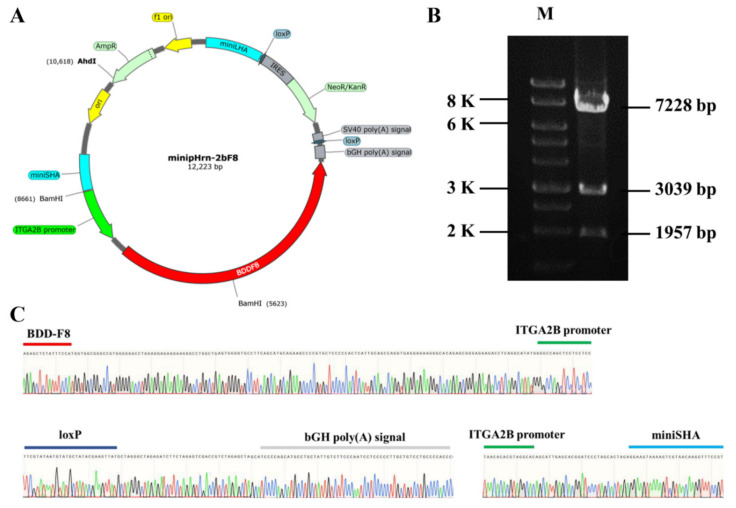
Construction of nonviral targeting vector minipHrn-2bF8. (**A**) The structure of the minipHrn-2bF8. (**B**) The plasmid minipHrn-2bF8 was identified by restriction endonuclease (BamH1 and Ahd1) digestion. Agarose gel electrophoresis showed expected bands of 7228 bp, 3039 bp, and 1957 bp (M, 1 KB DNA ladder). (**C**) Sequencing analysis of truncated *ITGA2B* promoter (green) and *BDDF8* (red), loxP (dark blue) and bGH poly(A) signal (gray), and short homologous arm (light blue).

In this study, the donor plasmid minipHrn-2bF8 was constructed based on the plasmid minipHrneo described previously. Between the 5′ long homologous arm (935 bp) and the 3′ short homologous arm (591 bp), the 889 bp megakaryocyte-specific promoter of *ITGA2B* gene driving the *BDDF8* and a promoterless neomycin resistance (*Neo*) cassette flanked by locus of X (cross)-overinP1 (LoxP) sites were contained (Figure 1A). The *Neo* cassette contained an encephalomyocarditis virus internal ribosomal entry site (EMCV-IRES), which enabled *Neo* gene expression under the control of the endogenous RNA polymerase I (Pol I) promoter after homologous recombination, so that the gene-targeted hiPSCs could be enriched by this promoter trapping. The donor plasmid was identified via restriction endonuclease digestion (Figure 1B) and Sanger sequencing (Figure 1C).

### 2.2. Gene Targeting of F8 into the rDNA Locus of HA-iPSCs Using TALENickases

**Figure 2:**
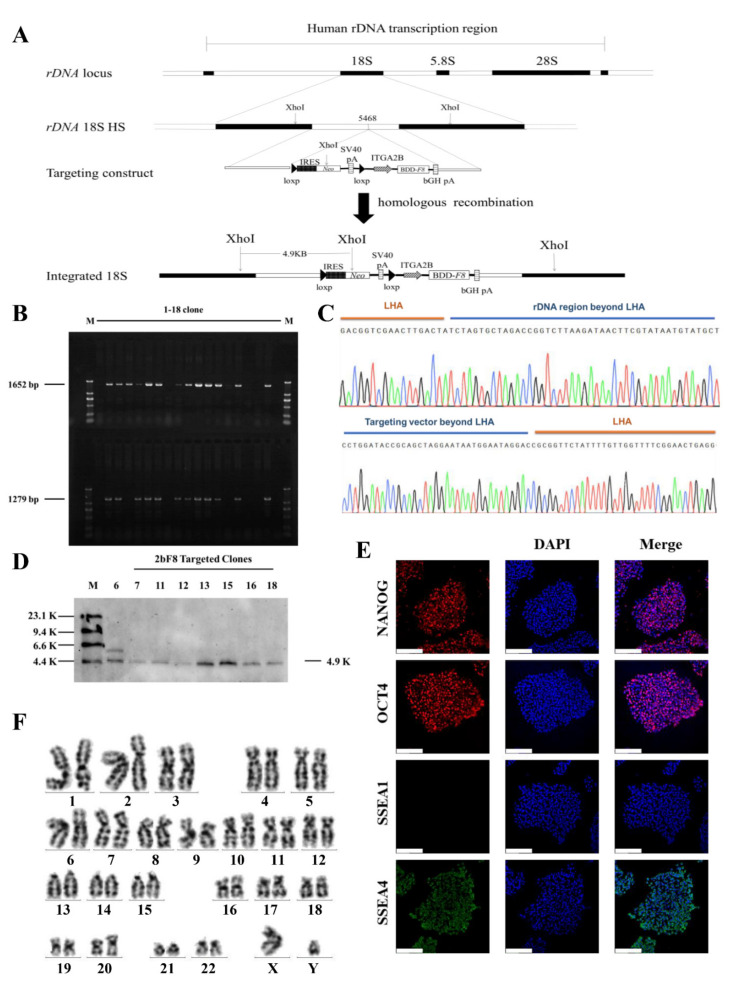
Gene targeting and screen. (**A**) The schematic diagram of gene targeting into the human rDNA locus. (**B**) Eighteen candidate targeted clones were picked and expanded, followed by genomic DNA extraction and PCR. The site-specific integrated clones obtained a 1652 bp band and a 1279 bp band with primers F2/R2 and F3/R3 (M, DL2000 DNA ladder). (**C**) The PCR products of F2/R2 were sequenced. (The peaks in each color represent specific bases, G: black, A: green, C: blue, T: red.) (**D**) Eight candidate integrated clones were randomly selected for Southern blot, and seven clones showed a specific target band of 4.9 K (M, DIG-labeled Molecular WeightII). (**E**) Immunofluorescence staining of 2bF8-iPSCs with NANOG, OCT4, SSEA1, and SSEA4, and 4′,6′-diamidino-2-phenylindole (DAPI) was used for staining the nuclei. Scale bar: 200 µm. (**F**) Karyotype of 2bF8-iPSCs.

The donor vectors minipHrn-2bF8 and TALENickases were nucleofected into the rDNA locus of HA-iPSCs (Figure 2A). After selection by G418 for 7 days, 18 clones were picked up and expanded. PCR screening with primers F2/R2 and F3/R3 showed that 12 out of 18 clones (66.67%) were positive for the expected band of 1652 bp and 1279 bp (Figure 2B). The PCR products were then verified by Sanger sequencing, which indicated the junction between the exogenous *2bF8* cassette and the endogenous rDNA sequence (Figure 2C). In addition, eight targeted clones were confirmed by Southern blotting using a probe homologous to the *Neo* gene, showing the gene-targeting fragments of 4920 bp (Figure 2D). We randomly selected one stable *F8*-modified hiPSCs (2bF8-iPSCs) for further research. These results indicated the site-specific integration of *2bF8* cassette into the rDNA locus. To analyze the off-target activity of TALENickases, gDNA of the HA-iPSCs and 2bF8-iPSCs were isolated and the top three potential off-target sites of TALENickases (https://tale-nt.cac.cornell.edu/) (accessed on 1 June 2021) were amplified, followed by Sanger sequencing, and no indels were observed compared to the HA-iPSCs (Figure S1).

Immunofluorescence showed that the 2bF8-iPSCs expressed the pluripotent markers NANOG, OCT4, and SSEA4, but did not express SSEA1 (Figure 2E). The 2bF8-iPSCs was karyotypically normal (Figure 2F).

### 2.3. Verification of F8 Expression in F8-Modified hiPSCs

**Figure 3:**
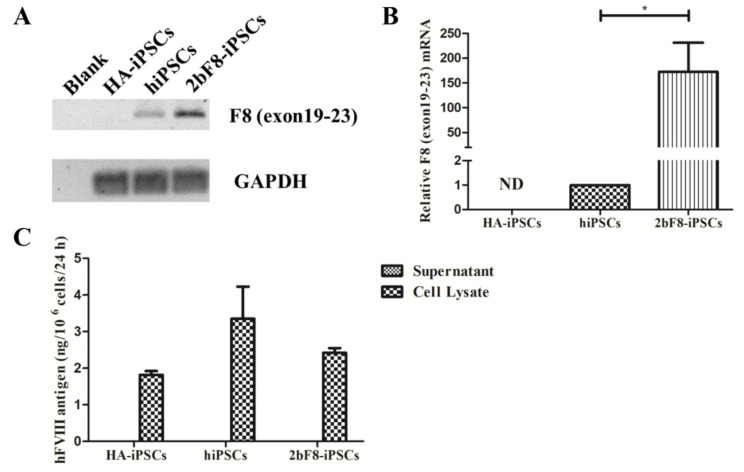
Verification of *F8* expression in *F8*-modified hiPSCs. (**A**) The mRNA of *F8* genes from HA-iPSCs, hiPSCs, and 2bF8-iPSCs were detected by RT-PCR. (**B**) Relative expression of *F8* in HA-iPSCs, hiPSCs, and 2bF8-iPSCs was detected by qRT-PCR. *GAPDH* was used as the internal reference. ND, not detected. * *p* < 0.05. Bars represent the mean ± SEM (*n* = 3, each group). (**C**) ELISA detection of the FVIII antigen in the supernatant and cell lysate of HA-iPSCs, hiPSCs, and 2bF8-iPSCs. Data represent the mean ± SEM (*n* = 3, independent cultures).

We designed specific primers based on the sequence crossing the exon 19-exon 23 of *F8* to detect the transcription of exogenous *F8* using RT-PCR. The results showed the transcripts crossing the exon 19-exon 23 of *F8* could be detected in 2bF8-iPSCs, but not in HA-iPSCs, suggesting that the transcript of *F8* exon 19-exon 23 in HA-iPSCs was nonexistent (Figure 3A). The qRT-PCR showed that the expression of integrated exogenous *F8* in the 2bF8-iPSCs was 172 times higher than that of endogenous *F8* in normal hiPSCs, indicating that *F8* mRNA could be effectively transcribed in 2bF8-iPSCs (Figure 3B). FVIII antigen was detected by ELISA in the cell lysates of HA-iPSCs (1.82 ng/10^6^ cells), 2bF8-iPSCs (2.42 ng/10^6^ cells), and hiPSCs (3.35 ng/10^6^ cells), while not detectable in the cell supernatant (Figure 3C).

### 2.4. Generation and Characterization of Induced HPCs (iHPCs) and Induced MKs (iMKs)

**Figure 4:**
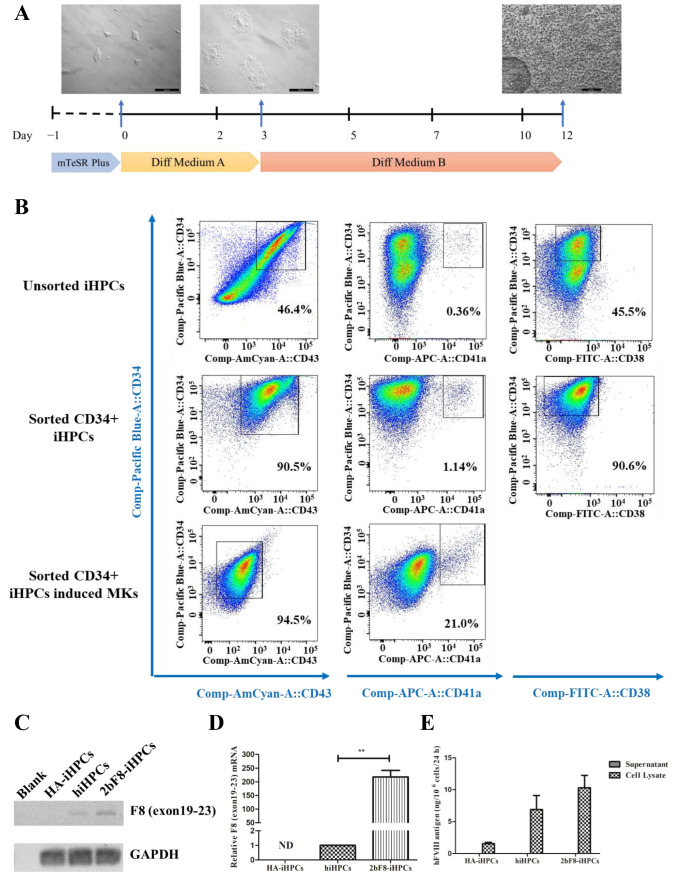
Generation and characterization of iHPCs and iMKs derived from hiPSCs and verification of *F8* expression in *F8*-modified iHPCs and iMKs. (**A**) Flow chart of the protocol for differentiation of hiPSCs into iHPCs and dynamic change in cellular morphology during the differentiation (day 0, 3, 12). (**B**) Flow cytometry analysis of iHPCs and iMKs derived from hiPSCs. (**C**) *F8* expression in HA-iHPCs, hiHPCs, and 2bF8-iHPCs according to RT-PCR. (**D**) Relative expression of *F8* in HA-iHPCs, hiHPCs, and 2bF8-iHPCs was detected by qRT-PCR. *GAPDH* was used as the internal reference. ND, not detected. ** *p* < 0.01. Bars represent the mean ± SEM (*n* = 3, each group). (**E**) FVIII antigen in the supernatant and cell lysate of HA-iHPCs, hiHPCs, and 2bF8-iHPCs was examined by ELISA. Data represent the mean ± SEM (*n* = 3, independent cultures).

We differentiated HA-iPSCs, 2bF8-iPSCs, and hiPSCs into HA-iHPCs, 2bF8-iHPCs, and hiHPCs, respectively, during which the cells converted from adherent to suspension (Figure 4A). After cell sorting with anti-CD34 beads, more than 90% of the iHPCs were double-positive for CD34 and CD43 and 90% were CD34 positive and CD38 negative, while only 47% of the unsorted iHPCs were double-positive for CD34 and CD43 and 45.5% were CD34 positive and CD38 negative. These results revealed that purer iHPCs were obtained via sorting. In addition, 1.1% of the sorted iHPCs showed as double-positive for CD34 and CD41a, indicating that sorted iHPCs have the potential to differentiate into iMKs.

Sorted iHPCs continued to differentiate into iMKs. After 5 days’ culture, the percentage of CD34 decreased and CD43 was negative, while the positive rate of CD41a increased to 2.1%, indicating that iMKs were obtained (Figure 4B).

### 2.5. Verification of F8 Expression in F8-Modified iHPCs

The qRT-PCR results showed that exogenous *F8* could be effectively expressed in the 2bF8-iHPCs (Figure 4C), and the *F8* expression of 2bF8-iHPCs was 218 times higher than that in hiHPCs (Figure 4D). Similar to that of hiPSCs, FVIII antigen was detected by ELISA in cell lysates, but not detected in the supernatants of iHPCs (Figure 4E). Therefore, it is indicated that the 2bF8-iHPCs produced abundant FVIII protein (10.31 ng/10^6^ cells) in cell lysates at the iHPCs stage, being prepared for the enrichment of FVIII in platelets.

### 2.6. Generation and Characterization of Induced MSCs (iMSCs)

**Figure 5:**
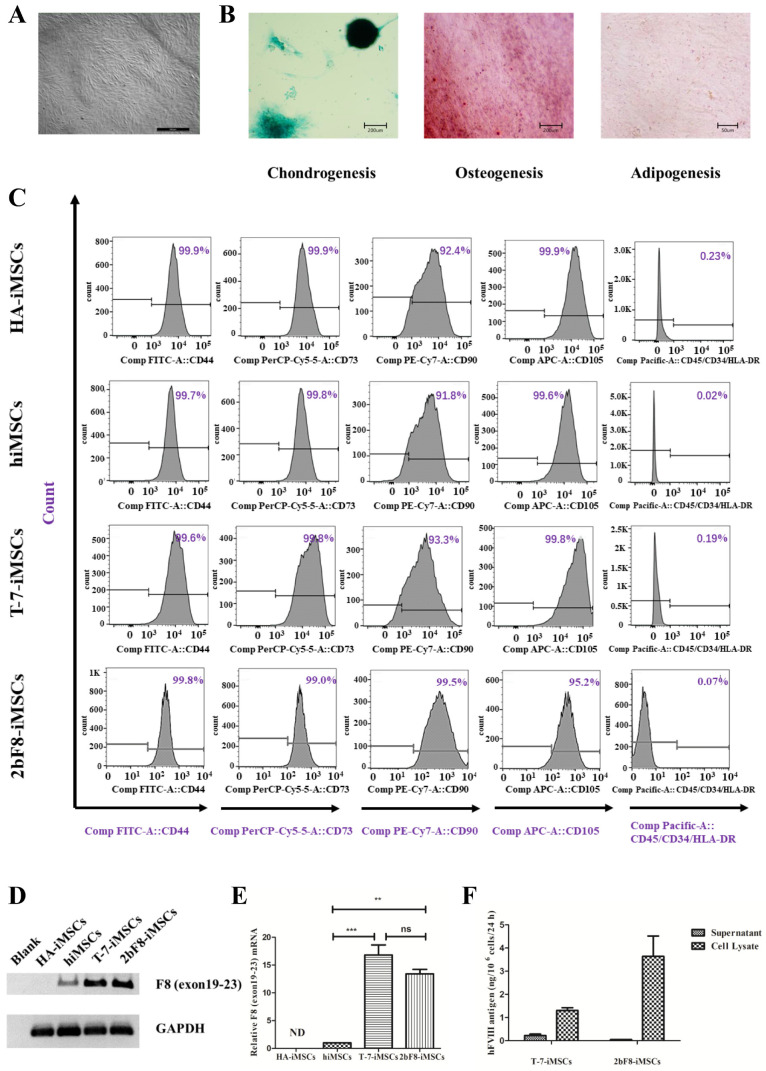
Generation and characterization of iMSCs derived from hiPSCs and verification of FVIII expression in *F8*-modified iMSCs. (**A**) Morphology of 2bF8-iMSCs differentiated from 2bF8-iPSCs. (**B**) Identification of osteogenic, chondrogenic, and adipogenic differential potential of 2bF8-iMSCs. (**C**) Flow cytometry analysis of iMSCs. (**D**) *F8* expression of HA-iMSCs, normal hiMSCs, and 2bF8-iMSCs was detected by RT-PCR. (**E**) Relative expression of *F8* in HA-iMSCs, hiMSCs, and 2bF8-iMSCs was detected by qRT-PCR. *GAPDH* was used as the internal reference. ND, not detected; ** *p* < 0.01; *** *p* < 0.001; ns, not significant. Bars represent the mean ± SEM (*n* = 3, each group). (**F**) The human FVIII antigen in the supernatant and cell lysate of HA-iHPCs, hiHPCs, and 2bF8-iHPCs was examined by ELISA. Data represent the mean ± SEM (*n* = 3, independent cultures).

Considering the difficulty in proliferation of iHPCs in vitro, MSCs were used as an alternative cell type to obtain sufficient cell sources. We differentiated 2bF8-iPSCs into 2bF8-iMSCs. HA-iMSCs, hiMSCs, and T-7-iMSCs (iMSCs derived from hiPSCs with *Cytomegalovirus* promoter (CMV)-driven *BDDF8*) were obtained in our previous studies [20]. iMSCs derived from hiPSCs showed typical fibroblast-like morphology (Figure 5A). Multilineage potential tests showed that HA-iMSCs, 2bF8-iMSCs, and hiMSCs have osteogenic, chondrogenic, and adipocytic differentiation abilities (Figure 5B). All iMSC lines were positive for CD44, CD73, CD90, and CD105, and were negative for CD34, CD45, and HLA-DR (Figure 5C), which was consistent with a previous report [21]. These results indicated that the hiPSCs were efficiently differentiated into iMSCs.

### 2.7. Verification of FVIII Expression in F8-Modified iMSCs

The exogenous *F8* expression was detected in the 2bF8-iMSCs via RT-PCR (Figure 5D). The *F8* mRNA of 2bF8-iMSCs was 13 times higher than that of hiMSCs, while the *F8* mRNA of T-7-iMSCs was 17 times higher than that of hiMSCs (Figure 5E), indicating that the universal promoter (CMV) was stronger than the megakaryocyte-specific promoter at the iMSCs stage. We evaluated the FVIII antigen in T-7-iMSCs and 2bF8-iMSCs by ELISA (Figure 5F) and found that FVIII in the supernatant of T-7-iMSCs was 0.23 ng/10^6^ cells and 0.05 ng/10^6^ cells in 2bF8-iMSCs, while it was opposite in the cell lysates of T-7-iMSCs (1.31 ng/10^6^ cells) and 2bF8-iMSCs (3.64 ng/10^6^ cells), providing the potential of FVIII stored in platelets derived from 2bF8-iMSCs.

## 3. Discussion

Immune response which results in the development of anti-FVIII or IX inhibitors with ineffectiveness of the substitutive therapy is still a key problem in hemophilia therapy. Heterotopic storage of FVIII in platelets is an ingenious method for HA gene therapy. This strategy can not only avoid the inhibitory antibodies to FVIII in circulation, but also increase the concentration of FVIII in the bleeding sites through platelet aggregation and achieve the goal of maximizing the therapeutic effect with low-level FVIII. Du et al. used a lentiviral vector encoding the *ITGA2B* gene promoter-driven *BDDF8* to transfect the isolated HSCs, then the cells were transplanted into a dog model and the platelet-specific human FVIII was detected, stored, and released from activated platelets [22]. In the present study, the truncated megakaryocyte-specific promoter was used to drive the expression of *BDDF8* that was targeted at the rDNA locus of HA-iPSCs. We detected the specificity of the promoter and found that the endogenous *ITGA2B* gene could be generally transcribed in hiPSCs and iHPCs, decreasingly transcribed in iMSCs, and not transcribed in T-lymphocyte (Figure S2), which was consistent with the results of the expression of *BDDF8* driven by this truncated promoter in corresponding cells and in the previous report.

The hiPSCs have been widely used as the target cells of gene editing due to their unlimited proliferation and multi-differentiation potential ability [23,24]. Gene editing in hiPSCs has been proven to be easily and effectively operated [25]. The hematopoietic capabilities of HPCs derived from gene-edited hiPSCs provides new opportunities for cell sources of hematopoietic transplantation, which is one of the most common cell transplantation approaches [26,27]. In this study, we differentiated *2bF8* gene-modified hiPSCs into iHPCs in vitro and detected FVIII expression. However, the number of iHPCs obtained by in vitro differentiation is limited, and it is still difficult to conduct in vivo experiments. Further studies need to be performed to improve the efficiency of differentiation of hiPSCs to HPCs and overcome the obstacles to clinical application.

In addition to aiming at obtaining platelets through HPCs differentiation, we tried to explore some alternative approaches [28,29]. Megakaryocytogenesis and platelet formation are the result of the interaction between HPCs, humoral factors, and bone marrow MSCs in a physiological environment. Human adipose-derived mesenchymal stromal/stem cells (ASCs) can differentiate into megakaryocyte lineages without gene modification and, besides that, ASCs secrete endogenous thrombopoietin to promote platelet production [19]. Since ASCs are composed of heterogenous cells, studies have established ASCs lines that extend to obtain a more homogenous population [30]. For HA patients, isolation of autologous ASCs is invasive. In the present study, we generated and gene-modified hiPSCs from the urine cells that were collected from HA patients non-invasively as the source for iMSCs expressing FVIII. These iMSCs could be continuously produced due to the infinite proliferation capacity of hiPSCs. For avoiding cell heterogeneity, iMSCs derived from hiPSCs are consistent in quantity and quality. A comparative study on the proliferation and differentiation abilities between iMSCs and autologous tissue-derived MSCs showed that iMSCs have better potential in adipogenic differentiation experiments in vitro [31]. Given the above, it is feasible to generate megakaryocytes from hiPSCs-derived iMSCs.

In summary, a megakaryocyte-specific promoter-driven *BDDF8* was efficiently targeted into the multi-copy rDNA locus of HA-iPSCs using a non-viral vector and TALENickases without potential off-target effects. The exogenous FVIII was stably expressed in iHPCs and iMSCs derived from *F8*-modified hiPSCs. These results provide a proof of concept for an efficient approach for the site-specific gene addition in hiPSCs for platelet-targeted HA gene therapy.

## 4. Materials and Methods

### 4.1. Cell Culture

The normal hiPSCs (DYR0100) were purchased from ATCC. HA-iPSCs [32] and T-7-iPSCs [33] were generated previously. Briefly, HA-iPSCs were reprogrammed by urine cells from a severe HA patient with *F8* intron 22 inversion. T-7-iPSCs were obtained by site-specific integration of CMV-driven *BDDF8* at the rDNA locus in HA-iPSCs. All hiPSCs were routinely cultured (37 °C, 5% CO_2_) on Matrigel- (BD Biosciences, Franklin Lakes, NJ, USA) coated 12-well plates (Corning, New York, NY, USA) in mTesR Plus medium (StemCell Technologies, Vancouver, BC, Canada).

The iMSCs were routinely cultured (37 °C, 5% CO_2_) on 0.1% gelatin (StemCell Technologies, Vancouver, BC, Canada) coated 10-cm dishes (Corning, New York, NY, USA) in αMEM basal medium (Thermo Fisher Scientific, Waltham, MA, USA) containing 10% FBS (Gibco, New York, NY, USA), 2 mM GlutaMAX^TM^ (Gibco, New York, NY, USA), and 0.1% bFGF (Gibco, New York, NY, USA).

### 4.2. Plasmids and Gene Targeting

In our previous study, we constructed the non-viral human rDNA targeting vector, minipHrneo [34,35]. In this study, we used two Phanta Max Master Mixes (Vazyme, Nanjing, China) and amplified the 889 bp promoter region upstream of *ITGA2B* gene from human genomic DNA (gDNA) with the following pairs of primers: F1: 5′-GGATCCGTGCTCAATGCTGTGCCTACGTG-3′/R1: 5′-GCTAGCTAGACATATGGGGCCAGCTCCTCCTCCTT-3′. We then inserted them into the targeting vector minipHrneo after digestion by NheI endonuclease (New England BioLabs, Hitchin, England) and BamH1 endonuclease (New England BioLabs, Hitchin, England). Then the *BDDF8* open reading frame was amplified from pHrnF8 vector [36] and inserted into the NheI restriction site of the targeting vector containing the 889 bp *ITGA2B* promoter by ClonExpress II One Step Cloning Kit (Vazyme, Nanjing, China) to generate the donor vector minipHrn-2bF8.

The HA-iPSCs were nucleofected with the Human Stem Cell Nucleofector^®^ Kit 2 (LONZA, Walkersville, MD, USA) according to the manufacturer’s instructions. Briefly, the hiPSCs were dissociated into single cells with TrypLE™ Express (Thermo Fisher Scientific, Waltham, MA, USA) and counted. For 1 × 10^6^ cells, 3 µg of each TALENickases plasmid [21] and 5 µg of donor vector minipHrn-2bF8 were used, and then the transfected cells were seeded on Matrigel-coated six-well plates in mTesR Plus medium with 10 µM of Y27632. Two days later, G418 (Sigma-Aldrich, St. Louis, MO, USA) was used for selection at the final concentration of 50 µg/mL. Then the cells were detached with TrypLE™ Express and 1000 cells were seeded on Matrigel-coated 6-cm dishes, and cultured in mTesR Plus medium with ClonR (STEMCELL Technologies, Vancouver, BC, Canada). After 9 to 11 days, clones were picked, expanded, and screened by PCR with the screen primers: F2: 5′-CCTGAGAAACGGCTACCACA-3′/R2: 5′-GAACTGCTTCCTTCACGACAT-3′ and F3: 5′-GGCAAGGAGATTGGGGATAA-3′/R3: 5′-CCAGACGAGACAGCAAACGG-3′. To analyze the off-target activity of TALENickases, targeted clones were detected via PCR, followed by Sanger sequencing. (OTS1F: 5′-GGGGCTGATGGGTTCCTAAAG-3′/OTS1R: 5′-CAGAAGAGTGCAAATGGTGAAGAAACACG-3′, OTS2F: 5′-CCCGGCCTTCCCCTAATTTC-3′/OTS2R: 5′-ATGTGGGTGGAGACGGAGCA-3′, and OTS3F: 5′-GAAGGGAGTCACTTCCATCTCTCA-3′/OTS3R: 5′-GTGGCTCTGAAATGCAGTGGC-3′).

### 4.3. RT-PCR and qRT-PCR

Total RNA was extracted using TRIzol reagent (Sigma-Aldrich, St. Louis, MO, USA) and RNA was treated with gDNA wiper mix (Vazyme, Nanjing, China) to eliminate genomic DNA. Then, RNA samples were reverse transcribed using Hiscrip R II Q RT Supermix (Vazyme, Nanjing, China). Primers based on exon 19 and exon 23 (F4: 5′-TCAACTCCATGCGAAGAGTG-3′/R4: 5′-GCTGGGATGAGCACACTTTT-3′) were used to detect the transcript of the *F8* gene. The glyceraldehyde-3-phosphate dehydrogenase (*GAPDH*) gene was used as an endogenous control.

### 4.4. Southern Blotting

The gDNA was extracted and digested with XhoI endonuclease (New England Biolabs, Hitchin, England) at 37 °C overnight and 10 µg of each digested sample was loaded on 1% agarose gel, followed by electrophoresis at 150 V for 2 h. The samples were then transferred to nylon membranes and the DIG-labeled Molecular WeightII (Roche, Basel, Switzerland) was used as a DNA marker. Then the membranes were hybridized with DIG Easy Hyb Granules (Roche, Basel, Switzerland) at 42 °C overnight, washed, and detected with CDP-Star. The probes were labeled by DIG Probe Synthesis Kit with F5: 5′-GCCGAGAAAGTATCCATCA-3′/R5: 5′-CAGAGTCCCGCTCAGAAG-3′. All reagents and materials were purchased from Roche.

### 4.5. Karyotyping

The hiPSCs were treated with 0.08 μg/mL colcemid (Sigma-Aldrich, St. Louis, MO, USA) for 2.5 h. Then the cells were trypsinized to single cells and incubated in 0.075 M KCl for 30 min at 37 °C. After fixing with Carnoy’s fixative (methanol: acetic acid = 3:1, Sinopharm Chemical Reagent Co., Ltd., Shanghai, China), metaphase chromosome spreads were prepared using air drying.

### 4.6. Immunofluorescence Staining

Cells plated on chamber slides were rinsed by DPBS (Thermo Fisher Scientific, Waltham, MA, USA), then fixed with 4% paraformaldehyde for 15 min, followed by permeabilization with PBST (DPBS containing 0.1% Triton X-100 (Sigma-Aldrich, St. Louis, MO, USA)) for 15 min. After washing with DPBS, cells were blocked in 5% BSA (R&D Systems, MN, USA) for 30 min, then incubated with the diluted primary antibodies (BD Biosciences, Franklin Lakes, NJ, USA) in 5% BSA at 4 °C overnight. After a thorough washing, the cells were blocked with 5% BSA for 30 min again and then incubated with the secondary antibodies for 1 h at room temperature in the dark. Nuclei were stained with DAPI (Sigma-Aldrich, St. Louis, MO, USA) for 5 min. Confocal (Leica, Wetzlar, Germany) photography was used to analyze the stained cells.

### 4.7. Differentiation of hiPSCs into iHPCs and iMKs

The hiPSCs, HA-iPSCs, and 2bF8-iPSCs were differentiated into hiHPCs, HA-iHPCs, and 2bF8-iHPCs using a STEMdiffTM hematopoietic kit (STEMCELL Technologies, Vancouver, BC, Canada) according to the manufacturer’s instructions. Briefly, cells were dissociated to aggregates by 0.5 mM EDTA (Thermo Fisher Scientific, Waltham, MA, USA) and plated 40–80 aggregates/well on Matrigel-coated six-well plates in mTesR Plus. The cells were cultured in differentiation A medium on the second day, then the medium was half-refreshed. On day 3, the cells were cultured with differentiation B medium and then the medium was half-refreshed every other day, and cells were gradually suspended. The iHPCs were harvested after being cultured in differentiation B medium for 7 days.

The differentiated cells were sorted using the Direct CD34 Progenitor Cell Isolation Kit (Miltenyi Biotec, Bergisch Gladbach, Germany) according to the manufacturer’s instructions.

The sorted CD34 positive iHPCs were cultured in StemSpan™ Serum-Free Expansion Medium (STEMCELL Technologies, Vancouver, BC, Canada) contained with StemSpan™ Megakaryocyte Expansion Supplement (STEMCELL Technologies, Vancouver, BC, Canada) to differentiate into megakaryocyte on low-attachment surface plates (Corning).

### 4.8. Characterization of iHPCs and iMKs

The iHPCs were dissociated into single cells with TrypLE™ Express and counted, and 5 × 10^4^ cells were suspended in 100 μL DPBS with 5% BSA. Then the cells were incubated with BV421-conjugated anti-human CD34, FITC-conjugated CD38, BV510-conjugated CD43, and APC-conjugated CD41a (BD Biosciences, Franklin Lakes, NJ, USA) at 4 °C for 30 min in the dark. The stained cells were washed twice using DPBS and characterized by flow cytometry. The iMKs were incubated with BV421-conjugated anti-human CD34, BV510-conjugated CD43, and APC-conjugated CD41a (BD Biosciences, Franklin Lakes, NJ, USA) at 4 °C for 30 min in the dark and analyzed by flow cytometry.

### 4.9. Differentiation of hiPSCs into iMSCs

A STEMdiffTM mesenchymal progenitor kit (STEMCELL Technologies, Vancouver, BC, Canada) was used to differentiate hiPSCs into iMSCs. According to the manufacturer’s protocol, hiPSCs were seeded on a Matrigel-coated 12-well plate at 5 × 10^4^ cells/cm^2^ in mTesR Plus containing 10 µM Y27632. After 2 days, the medium was refreshed with STEMdiffTM-ACF mesenchymal induction medium (STEMCELL Technologies, Vancouver, BC, Canada) for 4 days. Then the cells were cultured in STEMdiffTM-ACF medium for an additional 2 days and passaged on a six-well plate precoated with STEMdiffTM-ACF attachment substrate (STEMCELL Technologies, Vancouver, BC, Canada). The cells were passaged every 3 days. After three passages, the cells were seeded onto a 0.1% gelatin-coated 10-cm dish in αMEM basal medium containing 10% FBS, 2 mM GlutaMAXTM, and 0.1% bFGF. The medium was changed every day.

### 4.10. Characterization and Identification of Differentiation Potential of iMSCs

The iMSCs were dissociated into single cells with TrypLE™ Express and counted, and 5 × 10^4^ cells were suspended in 100 μL DPBS with 5% BSA. Then cells were incubated with BB515-conjugated CD44, Precp-Cy5.5-conjugated CD73, PE-Cy7-conjugated anti-human CD90, APC-conjugated CD105, BV421-conjugated anti-human CD34, CD45, and HLA-DR (BD Biosciences, Franklin Lakes, NJ, USA) at 4 °C for 30 min in the dark. The stained cells were washed twice in DPBS and characterized by flow cytometry (BD Biosciences, Franklin Lakes, NJ, USA).

The differentiation potential of iMSCs was identified via osteogenesis (Gibco, New York, NY, USA), adipogenesis (STEMCELL Technologies, Vancouver, BC, Canada), and chondrogenesis (STEMCELL Technologies, Vancouver, BC, Canada) differentiation kits, according to the manufacturer’s instructions. After being cultured with differentiation medium for 2 to 3 weeks, cells were stained with alizarin red, oil red O, or alcian blue dye (Cyagen Biosciences Inc., Guangzhou, China)) for 30 min separately, then the stained cells were analyzed using a light microscope.

### 4.11. FVIII Assay

The hiPSCs and iMSCs culture supernatants were harvested 24 h after the medium was changed. The cells were isolated by TrypLE^TM^ Express, counted, and re-suspended in a 600 µL sample dilution (Cedarlane, Burlington, ON, Canada). The iHPCs culture supernatants and cells were harvested by centrifugation 24 h after the medium was changed. The cells were counted and re-suspended in a 600 µL sample dilution (Cedarlane, Burlington, ON, Canada). The cells were lysed by three freeze–thaw cycles and the supernatant was collected by centrifugation. ELISA was performed using paired antibodies for ELISA-factor VIII:C (Cedarlane, Burlington, ON, Canada) according to the manufacturer’s instructions.

### 4.12. Statistical Analysis

GraphPad Prism 5.0 was used for data analysis. Data were analyzed using ANOVA for more than two groups. All values are presented as the mean ± SEM.

## 5. Conclusions

The *BDDF8* gene driven by a megakaryocyte-specific promoter was efficiently targeted into the rDNA locus of HA-iPSCs and stable expression of exogenous FVIII in *F8*-modified iHPCs and iMSCs was verified. This innovative strategy based on platelets derived from gene-modified hiPSCs can provide a promising alternative for HA gene therapy.

## Data Availability

All data supporting the reported result in this study can be found in the Supplementary Materials.

## Supplementary Materials

The following are available online at https://www.mdpi.com/article/10.3390/ijms23020623/s1.
