# Supplementary material for: Ectopic Expression of FVIII in HPCs and MSCs Derived from hiPSCs with Site-Specific Integration of ITGA2B Promoter-Driven BDDF8 Gene in Hemophilia A
Source: Int J Mol Sci. 2022 Jan 6;23(2):623. doi: 10.3390/ijms23020623 (PMC8775870; doi:10.3390/ijms23020623)
Supplement: Supplementary file 1 [file ijms-23-00623-s001.zip › ijms-1518359-supplementary.pdf]

Supplementary Materials

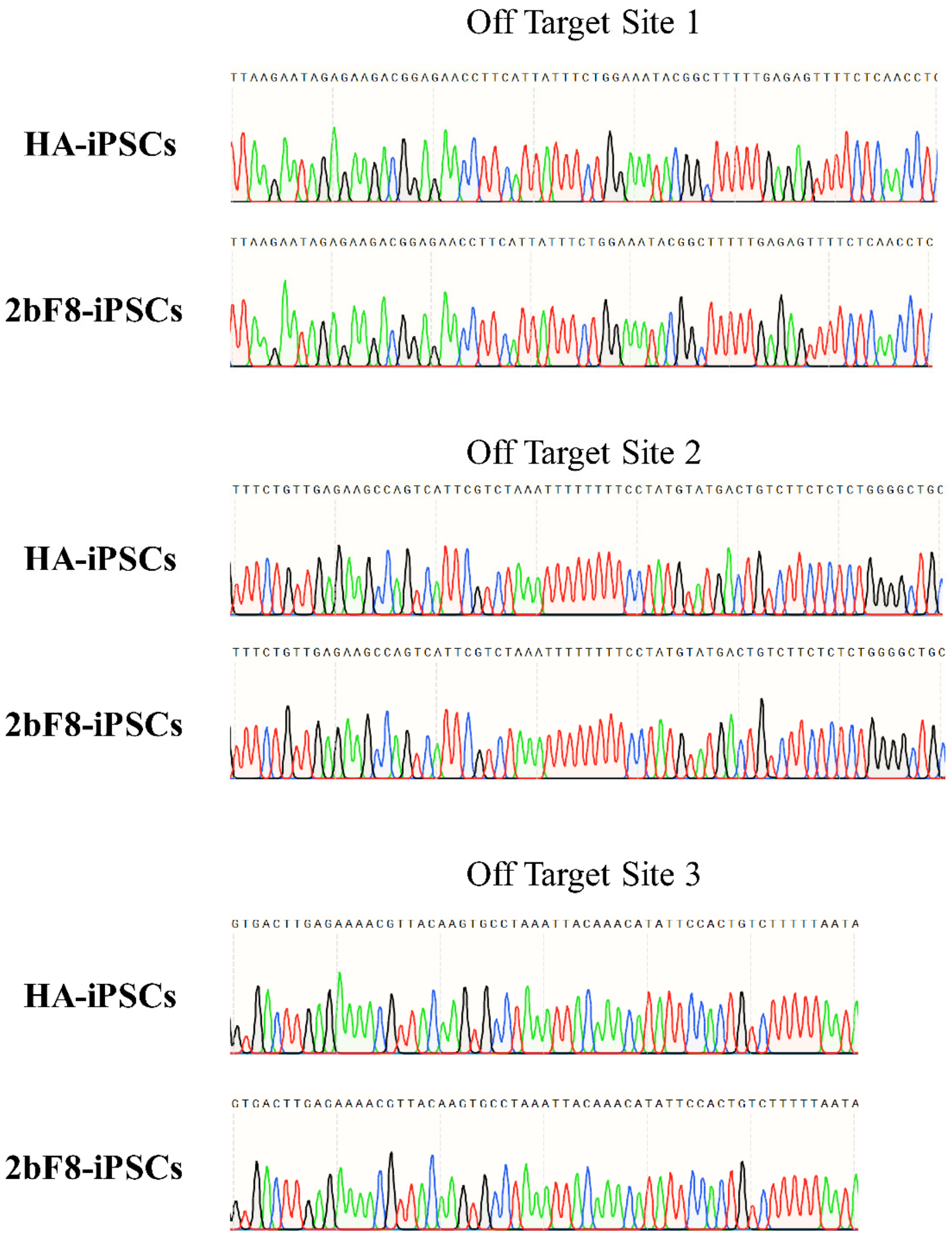

Figure S1. Three potential off-target sites were Sanger sequenced in patient iPSCs (HA-iPSCs) and targeted iPSCs (2bF8-iPSCs).

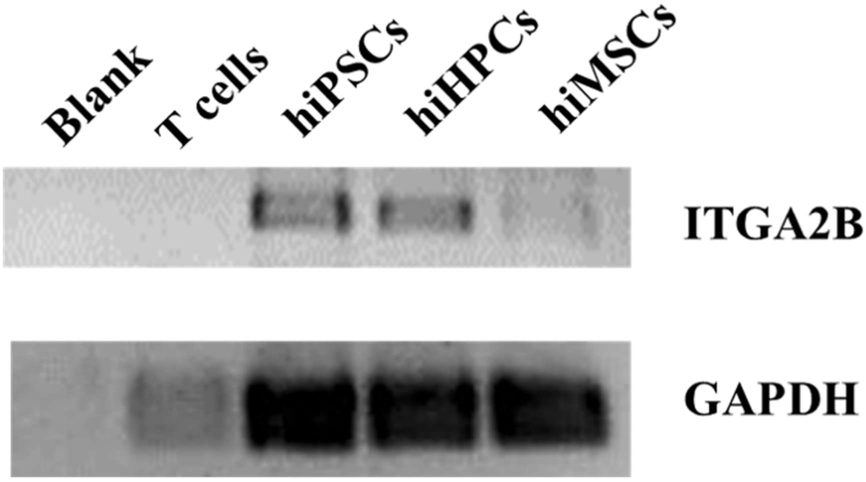

Figure S2. The endogenous *ITGA2B* gene expression. The endogenous *ITGA2B* expression from hiPSCs, hiHPCs, hiMSCs and human primary T cells were detected by RT-PCR.
